# Gasdermin C Is Upregulated by Inactivation of Transforming Growth Factor β Receptor Type II in the Presence of Mutated *Apc*, Promoting Colorectal Cancer Proliferation

**DOI:** 10.1371/journal.pone.0166422

**Published:** 2016-11-11

**Authors:** Masashi Miguchi, Takao Hinoi, Manabu Shimomura, Tomohiro Adachi, Yasufumi Saito, Hiroaki Niitsu, Masatoshi Kochi, Haruki Sada, Yusuke Sotomaru, Tsuneo Ikenoue, Kunitoshi Shigeyasu, Kohji Tanakaya, Yasuhiko Kitadai, Kazuhiro Sentani, Naohide Oue, Wataru Yasui, Hideki Ohdan

**Affiliations:** 1 Department of Gastroenterological and Transplant Surgery, Applied Life Sciences, Institute of Biomedical & Health Sciences, Hiroshima University, Hiroshima, Japan; 2 Department of Surgery, Institute for Clinical Research, National Hospital Organization Kure Medical Center and Chugoku Cancer Center, Hiroshima, Japan; 3 Natural Science Center for Basic Research and Development, Hiroshima University, Hiroshima, Japan; 4 Division of Clinical Genome Research, the Institute of Medical Science, University of Tokyo, Tokyo, Japan; 5 Department of Surgery, National Hospital Organization Iwakuni Clinical Center, Yamaguchi, Japan; 6 Departments of Gastroenterology and Metabolism, Applied Life Sciences, Institute of Biomedical & Health Sciences, Hiroshima University, Hiroshima, Japan; 7 Department of Health Science, Program in Human Culture and Science, Prefectural University of Hiroshima, Hiroshima, Japan; 8 Department of Molecular Pathology, Applied Life Sciences, Institute of Biomedical & Health Sciences, Hiroshima University, Hiroshima, Japan; University of Kansas School of Medicine, UNITED STATES

## Abstract

Mutations in *TGFBR2*, a component of the transforming growth factor (TGF)-β signaling pathway, occur in high-frequency microsatellite instability (MSI-H) colorectal cancer (CRC). In mouse models, *Tgfbr2* inactivation in the intestinal epithelium accelerates the development of malignant intestinal tumors in combination with disruption of the Wnt-β-catenin pathway. However, no studies have further identified the genes influenced by *TGFBR2* inactivation following disruption of the Wnt-β-catenin pathway. We previously described *CDX2P-G19Cre*;*Apc*^*flox/flox*^ mice, which is stochastically null for *Apc* in the colon epithelium. In this study, we generated *CDX2P-G19Cre*;*Apc*^*flox/flox*^;*Tgfbr2*^*flox/flox*^ mice, with simultaneous loss of *Apc* and *Tgfbr2*. These mice developed tumors, including adenocarcinoma in the proximal colon. We compared gene expression profiles between tumors of the two types of mice using microarray analysis. Our results showed that the expression of the murine homolog of *GSDMC* was significantly upregulated by 9.25-fold in tumors of *CDX2P-G19Cre*;*Apc*^*flox/flox*^;*Tgfbr2*^*flox/flox*^ mice compared with those of *CDX2P-G19Cre*;*Apc*^*flox/flox*^ mice. We then investigated the role of GSDMC in regulating CRC tumorigenesis. The silencing of GSDMC led to a significant reduction in the proliferation and tumorigenesis of CRC cell lines, whereas the overexpression of GSDMC enhanced cell proliferation. These results suggested that GSDMC functioned as an oncogene, promoting cell proliferation in colorectal carcinogenesis. In conclusion, combined inactivation of both Apc and Tgfbr2 in the colon epithelium of a CRC mouse model promoted development of adenocarcinoma in the proximal colon. Moreover, GSDMC was upregulated by *TGFBR2* mutation in CRC and promoted tumor cell proliferation in CRC carcinogenesis, suggesting that GSDMC may be a promising therapeutic target.

## Introduction

The classic paradigm of colorectal cancer (CRC) formation follows the adenoma-carcinoma sequence, in which CRC begins as an adenoma [1]. Additionally, CRC can be classified into two predominant forms of genomic instability: chromosome instability (CIN) and microsatellite instability (MSI). The subclass of MSI accounts for about 15% of CRCs and results from dysfunction of the DNA mismatch repair system [2–5].

Transforming growth factor (TGF)-β signaling has the potential to function as a tumor suppressor and regulates various biological processes, including cell growth, differentiation, apoptosis, extracellular matrix modeling, and immune response [6]. TGF-β signal inactivation occurs in many cancers, including pancreatic, breast, and colorectal cancer. In CRC, the components of the TGF-β signaling pathway, specifically *TGFBR2* and *Smad4*, are frequently mutated [7, 8]. *TGFBR2* mutations occur in the latter phase of CRC carcinogenesis when adenoma transitions to carcinoma in approximately 60–90% of high-frequency microsatellite instability (MSI-H) CRCs [9–13]. In clinical studies examining how mutations in the *TGFBR2* gene affect the development of MSI-H CRCs, tumors with *TGFBR2* mutations were shown to be more frequently located in the right-sided colon, usually had a poor degree of differentiation, tended to appear more frequently as Dukes B stage, and had worse prognoses than those without mutations, indicating that *TGFBR2* mutations contributed to tumor progression through the MSI pathway [14]. However, MSI-H tumors with *TGFBR2* mutations have been shown to be associated with better prognoses in resected stage III CRCs [9] but similar prognoses to those without *TGFBR2* mutations in a population-based study [15]. Thus, the association between prognosis and *TGFBR2* mutations in MSI-H CRCs is unclear.

The effects of *TGFBR2* mutations in the intestinal epithelium in cancer formation have also been studied in several genetically engineered mouse models [16]. Although *Tgfbr2* inactivation alone does not cause tumor-related changes, *Tgfbr2* conditional knockout mouse models have indicated that *Tgfbr2* inactivation in the intestinal epithelium accelerates the development of malignant intestinal tumors in combination with mutations in *Apc* [17], *Kras* [18], or *Pten* [19]. Therefore, such *in vivo* studies have demonstrated that *TGFBR2* inactivation acts synergistically with other aberrant signaling pathways that are often deregulated in CRC, such as the Wnt-β-catenin, RAS-RAF, and phosphoinositol 3-kinase (PI3K) pathways, to promote tumor development. However, no *in vivo* studies have identified the genes influenced by *TGFBR2* inactivation in the context of Wnt-β-catenin signaling disruption during colon tumor formation. These previously established CRC mouse models exhibited tumors predominantly in the small intestine. However, to mimic CRC, the ideal model would exhibit tumors in the colon because cellular responses to TGF-β signaling depend on the cell type and physiological condition [20, 21]. Our prior studies had revealed that mice carrying transgenes regulated by a 9.5-kb fragment containing 5′-flanking sequences from the human CDX2 promoter (CDX2P9.5) showed tightly restricted transgene expression in the colon epithelium [22]. Moreover, *CDX2P9*.*5-G19Cre*;*Apc*^*flox/flox*^ mice exhibited bi-allelic *Apc* inactivation in the colon epithelium initiated with stochastic activation of Cre recombinase with 19 guanine nucleotides (G19Cre) introduced downstream of the initiating ATG codon, followed by a frameshift reversion mutation in mononucleotide repeats [23–25]. This mouse model developed many polypoid tumors, including noninvasive adenocarcinoma, in the proximal colon.

In this study, we generated *CDX2P-G19Cre*;*Apc*^*flox/flox*^;*Tgfbr2*^*flox/flox*^ mice lacking Tgfbr2 and Apc protein specifically in the colon epithelium. We then compared the comprehensive gene expression profiles of tumors from *Apc* mutant mice and *Apc*/*Tgfbr2* mutant mice using microarray analysis. This analysis allowed us to elucidate the mechanisms inducing the characteristics of CRC with *TGFBR2* mutation and identify genes that may act as biomarkers or therapeutic targets in CRCs harboring *TGFBR2* mutations.

## Materials and Methods

### Ethics statements

This study was performed in strict accordance with the Guide for the Care and Use of Laboratory Animals and the local committee for animal experiments. All animal protocols were approved by the Institutional Animal Care and Use Committee of Hiroshima University (Permit Number: A15-57). We checked the body weights of the mice every day, and euthanized them immediately after weight loss was detected. Surgery was performed under sodium pentobarbital anesthesia, and all efforts were made to minimize the suffering of the mice. Mice were euthanized by CO2 asphyxiation as per IACUC guidelines.

### Generation of mouse lines

*Tgfbr2*^*flox/flox*^ mice (C57BL/6J) provided by H Ijichi (Tokyo University) [26] were mated with *Apc*^*flox/flox*^ mice [580S, [27]] to generate *Apc*^*flox/flox*^;*Tgfbr2*^*flox/flox*^ mice. *Apc*^*flox/flox*^;*Tgfbr2*^*flox/flox*^ mice were then mated with *CDX2P9*.*5-G19Cre* mice [23] to generate *Apc*^*flox/+*^;*Tgfbr2*^*flox/+*^; *CDX2P-G19Cre* mice. Finally, *Apc*^*flox/+*^;*Tgfbr2*^*flox/+*^; *CDX2P-G19Cre* mice were interbred to obtain *CDX2P-G19Cre*;*Apc*^*flox/flox*^;*Tgfbr2*^*flox/flox*^ mice and *CDX2P-G19Cre*;*Apc*^*flox/flox*^ mice. All mice were housed under specific pathogen-free conditions. Teklad Mouse Breeder Diet 8626 (Harland-Teklad) and automatically supplied water were provided to all mice used in tumorigenesis experiments. The breeding room was maintained at a constant temperature of 23°C ± 2°C, relative humidity of 50% ± 5%, 15–20 air changes per hour, and a 12-h light/dark cycle, with lights on at 8:00 am. Four or five mice were housed per cage with chopped wood bedding. Genotyping of *Apc* and *Tgfbr2* alleles was performed with genomic DNA extracted from mouse tails, as described previously [24,26].

### Tissue harvesting and fixation

After necropsy, the gastrointestinal tracts of mice were removed and analyzed for macroscopically visible lesions. The tissues were washed with PBS containing 0.01% Triton X-100 at 4°C with agitation. For pathological analyses and tRNA extraction, one part of the tumor tissue was fixed in 4% paraformaldehyde and embedded in paraffin, and the other part was embedded in OCT compound (Sakura Finetek Japan, Tokyo, Japan), rapidly frozen, and stored in liquid nitrogen.

### Laser capture microdissection and gene expression profiling

Tissues were fixed in OCT compound, and frozen sections (12 μm) were then cut, dehydrated, and stained with hematoxylin. tRNA was extracted from cancer tissues dissected with a LMD6500 laser capture microdissection device (Leica MICROSYSTEMS, Tokyo, Japan), and samples with an RNA integrity number greater than 6.0 were analyzed further. Gene expression profiling was compared between *CDX2P-G19Cre*;*Apc*^*flox/flox*^;*Tgfbr2*^*flox/flox*^ (n = 3) and *CDX2P-G19Cre*;*Apc*^*flox/flox*^ (n = 3) mouse tumors with a Mouse Gene 1.0 ST Array (Affymetrix, Tokyo, Japan). The arrays were scanned using a GeneArray scanner (Affymetrix), and gene expression data were analyzed using GeneSpring GX software ver. 11 (Agilent Technology, Santa Clara, CA, USA). The robust multichip analysis (RMA) algorithm was used for normalization to remove artifactual differences between arrays, and cut-off values were set at less than 20% to eliminate poorly reproducible entities between chips. Furthermore, gene set enrichment analysis (GSEA) of microarray data was performed to determine whether a priori-defined gene sets showed statistically significant concordant differences in gene expression between *CDX2P-G19Cre*;*Apc*^*flox/flox*^;*Tgfbr2*^*flox/flox*^ and *CDX2P-G19Cre*;*Apc*^*flox/flox*^ mouse tumors. The GSEA analyses were performed using GSEA2.2.2 software downloaded from the Molecular Signatures Database (MSigDB) and Simple Array Analyzer (developed by iAnalyze {Kagoshima, Japan}) according to the user manuals. The analysis was performed with the gene sets (c4: the computational gene set obtained by mining large cancer-related microarray data, and c5: Gene Ontology gene set) downloaded from MSigDB [28, 29].

### qRT-PCR

cDNA was generated using a QuantiTect Reverse Transcription Kit (Qiagen, Valencia, CA, USA) and was amplified with a Rotor-Gene Q 2PLEX HRM Real-Time PCR system (Qiagen). The qPCR mixture was prepared in a final volume of 25 μL, including 2× Rotor-Gene SYBR Green PCR Mix (Qiagen), 10 μM forward/reverse primers, and 32 ng cDNA. The amplification protocol consisted of denaturation at 95°C for 5 min, followed by 40 cycles of 95°C for 5 s and 60°C for 10 s. Beta-2-microglobulin (*B2M*) was used as an internal control. The PCR primers used for gene analysis are shown in S1 Table.

### Immunohistochemistry

For mouse tissues, we carried out immunohistochemical staining of β-catenin and immunofluorescence staining of Tgfbr2 with a rabbit anti-mouse TGFβRII polyclonal antibody (sc-220; Santa Cruz Biotechnology, Dallas, TX, USA) as previously described [30, 31]. For human tissues, formalin-fixed, paraffin-embedded samples were sectioned to 5-μm-thick sections and stained for GSDMC using a Catalyzed Signal Amplification System (Dako Japan, Tokyo, Japan), which is based on streptavidin-biotin-horseradish peroxidase complex formation. After deparaffinization and rehydration, the sections were treated with target retrieval solution (pH 9.0) at 96°C for 40 min. A rabbit anti-human GSDMC polyclonal antibody (NBP1-91924; Novus Biologicals, Littleton, CO, USA) were used at a dilution of 1:100, followed by incubation with peroxidase-labeled anti-rabbit IgG for 60 min.

### Surgical specimens

We investigated GSDMC mRNA and protein expression in cancer and corresponding adjacent normal colonic tissues for 44 consecutive CRCs resected at the Hiroshima University Hospital (Hiroshima, Japan) from 2013 to 2014. Patient characteristics were collected from a prospective database, and CRC was staged according to the American Joint Commission for Cancer Staging (6th edition). Written informed consent for participation in the study was obtained from all participants. This study was approved by Ethical Committee for Epidemiology of Hiroshima University (Permit Number: Epidemiology-744).

### Cell lines and reagents

All cell lines were obtained from the American Type Culture Collection (ATCC) from 1998 to 2000. The amphotropic Phoenix packaging cell line was provided by G. Nolan (Stanford University, Palo Alto, CA, USA). Details of cell culture conditions were previously described [32].

### RNA interference

Two siRNA duplexes targeting GSDMC (GSDMC siRNA1 and siRNA2) and a nonsilencing siRNA duplex (MISSION siRNA Universal Negative Control: SIC-002; Sigma Aldrich, Hokkaido, Japan) were synthesized by Sigma Aldrich. Sequences of GSDMC siRNAs are listed in S2 Table. Cells were cultured in antibiotic-free medium for 24 h and then transfected with siRNAs (80 pmol) using Lipofectamine RNAiMAX Transfection Reagent (Life Technologies, Tokyo, Japan). The silencing effect of the siRNAs was examined by qRT-PCR 48 h after transfection.

### Plasmid construction

A 1606-bp fragment of the *GSDMC* allele containing a Flag tag at the 3′ end was amplified from cDNA of LoVo cells using the indicated primers (S2 Table) and then was inserted into the retroviral expression vector pDON-5 neo (TaKaRa, Shiga, Japan) by SalI/BglII digestion to form the vector pDON-5/GSDMC. All plasmids derived from PCR products were verified by sequencing.

The hairpin-loop oligonucleotides containing GSDMC siRNA2 and nonsilencing siRNA (S2 Table) were synthesized and inserted into pSUPER.retro.neo+gfp (OligoEngine, Seattle, WA, USA) by BglII/HindIII digestion to generate pSUPER/GSDMC shRNA2 and pSUPER/nonsilencing shRNA.

### Retroviral infections

Phoenix packaging cells were transfected with retroviral constructs; supernatants containing nonreplicating amphotropic virus were harvested. For GSDMC overexpression, SW480 and WiDr cells were infected with virus containing pDON-5/GSDMC and pDON-5 vectors. For GSDMC silencing, LoVo cells were infected with virus containing pSUPER/GSDMC shRNA2 and pSUPER/nonsilencing shRNA. Cells were selected with neomycin (500, 600, and 250 μg/mL, respectively for SW480, WiDr, and LoVo cells) for 2–3 weeks as previously described [32].

### Western blotting

Western blot analysis was performed essentially as previously described [32]. Anti-GSDMC rabbit polyclonal antibodies (TA315616; ORIGENE, CA, USA) and anti-β actin monoclonal antibodies (clone AC-15; Sigma Aldrich) were used at 1:1000 dilutions.

### Cell proliferation assays

Cell proliferation was measured using a CellTiter 96 AQueous One Solution Cell Proliferation Assay (MTS) kit (Promega KK, Tokyo, Japan). The target cells (700 cells/well) were seeding and incubated in a 96-well plate. MTS reagent (20 μL) was added to each well, and plates were incubated for 2 h in a humidified incubator at 37°C with an atmosphere containing 5% CO_2_ for 0, 48, 96, or 144 h after transfection. The absorbance (A) of each plate was measured at 490 nm. Assays were performed in triplicate.

### Soft agar colony formation assays

Colony formation assays were performed in 35-mm dishes as described previously [33, 34]. Assays were performed in triplicate.

### *In vivo* tumorigenesis assays

Female BALB/cA Jcl-nu mice (CLEA Japan, Tokyo, Japan) were used at 5 weeks of age. A total of 1.0 × 10^7^ LoVo cells stably expressing *GSDMC* shRNA2 or nonsilencing shRNA were subcutaneously injected into the right flanks of nude mice. The tumor size was measured by a Vernier caliper every 3 days from days 5–14 after cell implantation. The volume was calculated using the following formula: V = 0.5a × b^2^, where a is the long diameter, and b is the short diameter. The tumors were removed and weighed on day 14.

### Statistical analysis

All values are expressed as means ± standard deviations (SDs). The statistical significance of differences was determined by Mann-Whitney U tests, χ^2^ tests, unpaired t test or Fisher’s exact tests. Differences with *p* value of less than 0.05 were considered statistically significant. All statistical analyses were performed using JMP 10 software (SAS Institute Inc.).

## Results

### *CDX2P-G19Cre*;*Apc*^*flox/flox*^;*Tgfbr2*^*flox/flox*^ mice developed polypoid lesions, including well-differentiated adenocarcinoma, in the colon

**Fig 1:**
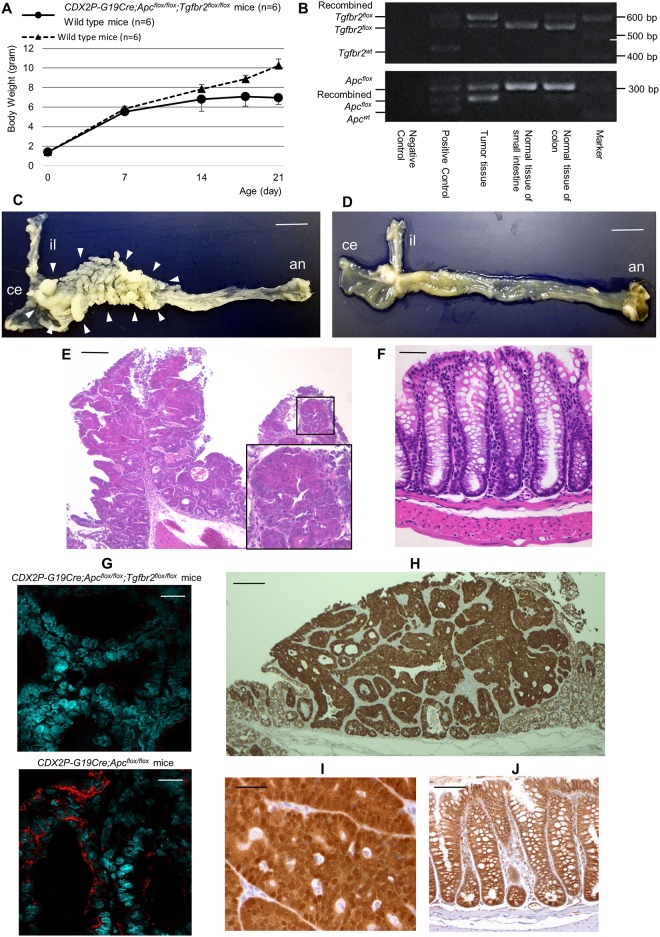
Tumorigenesis in *CDX2P-G19Cre*;*Apc*^*flox/flox*^;*Tgfbr2*^*flox/flox*^ mice and histological analysis of tumors. (A) Body weights of *CDX2P-G19Cre*;*Apc*^*flox/flox*^;*Tgfbr2*^*flox/flox*^ and control (wild-type) mice. Solid line, *CDX2P-G19Cre*;*Apc*^*flox/flox*^;*Tgfbr2*^*flox/flox*^ mice; dotted line, control mice. Points, means; bars, SDs. (B) Genotyping of *Apc* and *Tgfbr2* in tumor, normal jejunum, and colon tissues. (C) Images of dissected terminal ileums and colons of *CDX2P-G19Cre*;*Apc*^*flox/flox*^;*Tgfbr2*^*flox/flox*^ mice, showing multiple polypoid lesions in the proximal colon. Scale bar, 1 cm. (D) Images of dissected terminal ileum and colon of control (wild-type) mice. Scale bar, 1 cm. il, ileum; ce, cecum; an, anus. The locations of polypoid lesions are indicated by arrowheads. (E) Histological analysis of hematoxylin and eosin (H&E)-stained sections of tumors from *CDX2P-G19Cre*;*Apc*^*flox/flox*^;*Tgfbr2*^*flox/flox*^ mice showing well-differentiated adenocarcinoma, and higher-power H&E-stained sections of cancer tissue (bottom right). Bar, 100 μm. (F) Histological analysis of H&E-stained sections of normal colon from control (wild-type) mice. Bar, 10 μm. (G) Tgfbr2 immunofluorescence staining of tumors from *CDX2P-G19Cre*;*Apc*^*flox/flox*^;*Tgfbr2*^*flox/flox*^ mice and *CDX2P-G19Cre*;*Apc*^*flox/flox*^ mice. The nuclei are shown in green, and Tgfbr2 is shown in red. upper panel: intact Tgfbr2 was not detected in tumors from *CDX2P-G19Cre*;*Apc*^*flox/flox*^;*Tgfbr2*^*flox/flox*^ mice; lower panel: Tgfbr2 was detected in tumors in *CDX2P-G19Cre*;*Apc*^*flox/flox*^ mice. Bar, 10 μm. (H, I) Immunohistochemical staining for β-Catenin in tumors from *CDX2P-G19Cre*;*Apc*^*flox/flox*^;*Tgfbr2*^*flox/flox*^ mice. (J) Immunohistochemical staining for β-Catenin in normal epithelium from control (wild-type) mice. (H) Bar, 100 μm. (I, J) Bar, 20 μm.

The growth of *CDX2P-G19Cre*;*Apc*^*flox/flox*^;*Tgfbr2*^*flox/flox*^ mice, as measured by body weight, was inhibited relative to that of wild-type mice after 14 days (Fig 1A), and *CDX2P-G19Cre*;*Apc*^*flox/flox*^;*Tgfbr2*^*flox/flox*^ mice did not live for more than 4 weeks, similar to those observed in *CDX2P-G19Cre*;*Apc*^*flox/flox*^ mice. The death in two genetically engineering mice was assumed to be tumor-related, caused by tumor bleeding because there was no suggestive finding of the ileus, such as the intestinal dilatation on the oral side of the tumors. Genetic analysis showed evidence of recombination of the *Apc*^*flox*^ and *Tgfbr2*^*flox*^ alleles in the tumor tissue, but not in the normal jejunum tissue without Cre expression, as well as some recombination in the normal colonic tissue, including those in which Cre protein was expressed but macroscopic tumors did not arise (Fig 1B). Mice were euthanized at 3 weeks of age to evaluate the development of colon tumors. The proximal colons of *CDX2P-G19Cre*;*Apc*^*flox/flox*^;*Tgfbr2*^*flox/flox*^ mice exhibited multiple polypoid lesions, and histological analysis showed that these tumors were well-differentiated adenocarcinomas, similar to those observed in *CDX2P-G19Cre*;*Apc*^*flox/flox*^ mice (Fig 1C–1F). Both types of genetically engineered mice had distant metastases. Although the morphological phenotypes of the two mouse models did not differ, immunofluorescence staining revealed that intact Tgfbr2 was not expressed in the tumors of *CDX2P-G19Cre*;*Apc*^*flox/flox*^;*Tgfbr2*^*flox/flox*^ mice but was expressed in those of *CDX2P-G19Cre*;*Apc*^*flox/flox*^ mice (Fig 1G). Immunohistochemical staining for β-catenin in serial sections of the tumor indicated nuclear and cytoplasmic accumulation of β-catenin (Fig 1H–1J), suggesting that the tumors developed through abnormal activation of the Wnt-β-catenin pathway.

### Identification of candidate genes whose expression was altered in response to *Tgfbr2* mutation by microarray analysis

**Fig 2:**
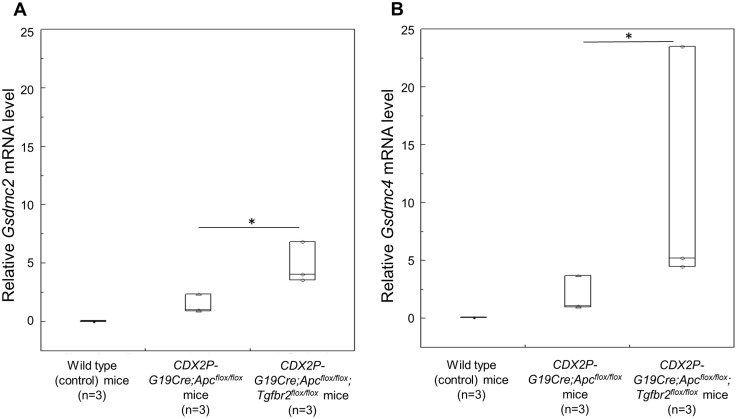
Quantitative reverse transcription PCR analysis. (A-B) Expression levels of *Gsdmc2* (A) and *Gsdmc4* mRNAs (B) in the tumors of *CDX2P-G19Cre*;*Apc*^*flox/flox*^;*Tgfbr2*^*flox/flox*^ and *CDX2P-G19Cre*;*Apc*^*flox/flox*^ mice, and in the normal proximal colon of wild-type (control) mice (n = 3 per group). *p < 0.05. Data are shown as box plots. The horizontal lines represent the median score, and the bottom and top of the boxes represent the 25th and 75th percentiles, respectively.

Differentially expressed genes were identified as those having a fold-change of at least 2.0 and a *p* value of less than 0.05 between two groups. Thirteen genes were significantly upregulated, and 37 genes were significantly downregulated in the tumors of *CDX2P-G19Cre*;*Apc*^*flox/flox*^;*Tgfbr2*^*flox/flox*^ mice compared with those of *CDX2P-G19Cre*;*Apc*^*flox/flox*^ mice (Table 1). The data discussed in this publication have been deposited in NCBI's Gene Expression Omnibus (Edgar *et al*., 2002) and are accessible through GEO Series accession number GSE82133 (http://www.ncbi.nlm.nih.gov/geo/query/acc.cgi?acc=GSE82133). Of these candidate genes, GasderminC2/GasderminC4 (*Gsdmc2|Gsdmc4)* was the most highly upregulated. Additionally, quantitative reverse transcription polymerase chain reaction (qRT-PCR) further confirmed the significant upregulation of *Gsdmc2|Gsdmc4* (Fig 2), thus validating the microarray results. The gene sets differentially expressed in tumors of *CDX2P-G19Cre*;*Apc*^*flox/flox*^;*Tgfbr2*^*flox/flox*^ mice in comparison with those of *CDX2P-G19Cre*;*Apc*^*flox/flox*^ mice as determined by GSEA software and Simple Array Analyzer are shown in S3 and S4 Tables.

**Table 1:** A selective list of genes that are differentially expressed (P < 0.05) in the tumors of *CDX2P-G19Cre*;*Apc*^*flox/flox*^;*Tgfbr2*^*flox/flox*^ mice compared with those of *CDX2P-G19Cre*;*Apc*^*flox/flox*^ mice with a fold-change of at least 2.0.

Gene description	Gene symbol	Fold^a^	P value^b^
**Genes whose expression was upregulated**			
gasdermin C4 | gasdermin C2	*Gsdmc4|Gsdmc2*	9.25	0.045
carbonyl reductase 2	*Cbr2*	3.75	0.034
gastrin releasing peptide receptor	*Grpr*	2.92	0.036
ectonucleotide pyrophosphatase/phosphodiesterase 3	*Enpp3*	2.60	0.039
Indian hedgehog	*Ihh*	2.43	0.007
SH3 domain containing ring finger 2	*Sh3rf2*	2.36	0.042
cytochrome P450, family 2, subfamily w, polypeptide 1	*Cyp2w1*	2.27	0.042
calcium/calmodulin-dependent protein kinase ID	*Camk1d*	2.11	0.046
annexin A13	*Anxa13*	2.11	0.035
secreted and transmembrane 1B	*Sectm1b*	2.05	0.019
phospholipase B domain containing 1	*Plbd1*	2.05	0.012
histone cluster 1, H1a	*Hist1h1a*	2.03	0.005
lumican	*Lum*	2.01	0.005
**Genes whose expression was downregulated**			
mucin 6, gastric	*Muc6*	4.86	0.001
prostate stem cell antigen	*Psca*	4.48	0.010
haptoglobin	*Hp*	4.26	0.001
protein tyrosine phosphatase, receptor type Z, polypeptide 1	*Ptprz1*	3.54	0.031
serum amyloid A 3	*Saa3*	3.20	0.030
clusterin	*Clu*	3.00	0.025
keratin 23	*Krt23*	2.82	0.028
U1b1 small nuclear RNA | U1b2 small nuclear RNA | U1b6 small nuclear RNA	*Rnu1b1|Rnu1b2|Rnu1b6*	2.82	0.050
neuronatin	*Nnat*	2.79	0.010
lipocalin 2	*Lcn2*	2.74	0.042
LIM and calponin homology domains 1	*Limch1*	2.61	0.037
ATP-binding cassette, sub-family B (MDR/TAP), member 1B	*Abcb1b*	2.47	0.005
glycosylphosphatidylinositol specific phospholipase D1	*Gpld1*	2.46	0.032
plasminogen activator, tissue	*Plat*	2.45	0.030
insulin-like growth factor 2	*Igf2*	2.41	0.046
predicted gene 22	*Gm22*	2.33	0.047
scavenger receptor class A, member 3	*Scara3*	2.29	0.002
synaptotagmin V	*Syt5*	2.28	0.031
dickkopf homolog 3 (Xenopus laevis)	*Dkk3*	2.23	0.018
villin-like	*Vill*	2.23	0.038
sidekick homolog 2 (chicken)	*Sdk2*	2.22	0.044
N-acetylated alpha-linked acidic dipeptidase-like 2	*Naaladl2*	2.21	0.031
ceruloplasmin	*Cp*	2.18	0.002
solute carrier family 22 (organic cation transporter), member 3	*Slc22a3*	2.18	0.037
epoxide hydrolase 1, microsomal	*Ephx1*	2.17	0.043
protocadherin beta 12	*Pcdhb12*	2.17	0.020
cyclin-dependent kinase inhibitor 1C (P57)	*Cdkn1c*	2.16	0.036
RNA U12, small nuclear	*Rnu12*	2.16	0.017
zinc finger protein 503	*Zfp503*	2.07	0.012
tenascin C	*Tnc*	2.05	0.036
solute carrier family 19 (sodium/hydrogen exchanger), member 3	*Slc19a3*	2.05	0.041
small nucleolar RNA, H/ACA box 74A	*Snora74a*	2.05	0.016
U3A small nuclear RNA	*Rnu3a*	2.04	0.011
CD1d1 antigen	*Cd1d1*	2.03	0.007
ring finger protein 180	*Rnf180*	2.02	0.004
family with sequence similarity 55, member D	*Fam55d*	2.02	0.011
WAP four-disulfide core domain 15B	*Wfdc15b*	2.01	0.017

^a^ Fold indicates a gene expression ratio of the tumors of *CDX2P-G19Cre*;*Apc*^*flox/flox*^;*Tgfbr2*^*flox/flox*^ mice to those of *CDX2P-G19Cre*;*Apc*^*flox/flox*^ mice.

^b^ P values were calculated by the unpaired t-test.

### GSDMC expression in human CRC

**Fig 3:**
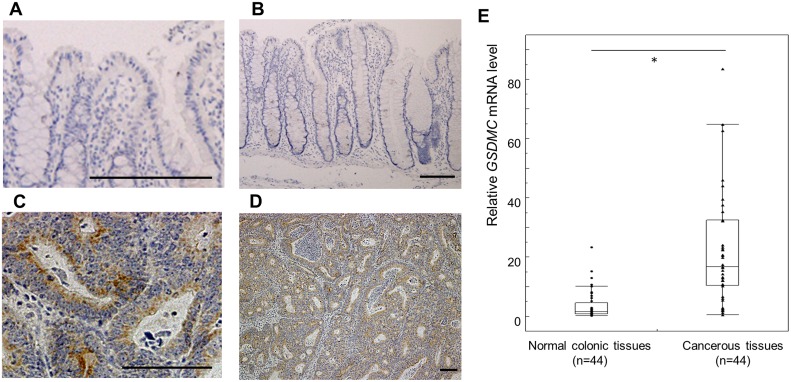
GSDMC immunohistochemical staining of normal colonic tissues (A, B) and CRC specimens (C, D). (A), (C) High-magnification image. (B), (D) Low-magnification image. GSDMC was not expressed in normal colonic tissues, whereas GSDMC was diffusely distributed in the cytoplasm of cancer cells. Scale bar 100 μm (E) The expression of *GSDMC* mRNA was significantly increased in cancer tissues compared with that in normal tissues. Data are shown as box plots. The horizontal lines represent the median score, the bottom and top of the boxes represent the 25th and 75th percentiles, respectively, and the whiskers represent the range of expression level.

Immunohistological analysis confirmed that GSDMC was not expressed in normal colonic tissues (Fig 3A and 3B), whereas GSDMC was expressed in the cancer tissues of almost all CRCs (Fig 3C and 3D). Consistent with these results, qRT-PCR analysis confirmed that *GSDMC* was upregulated in CRC tissues compared with that in corresponding adjacent normal colonic tissues (Fig 3E).

### Effects of GSDMC silencing and overexpression on cell growth

**Fig 4:**
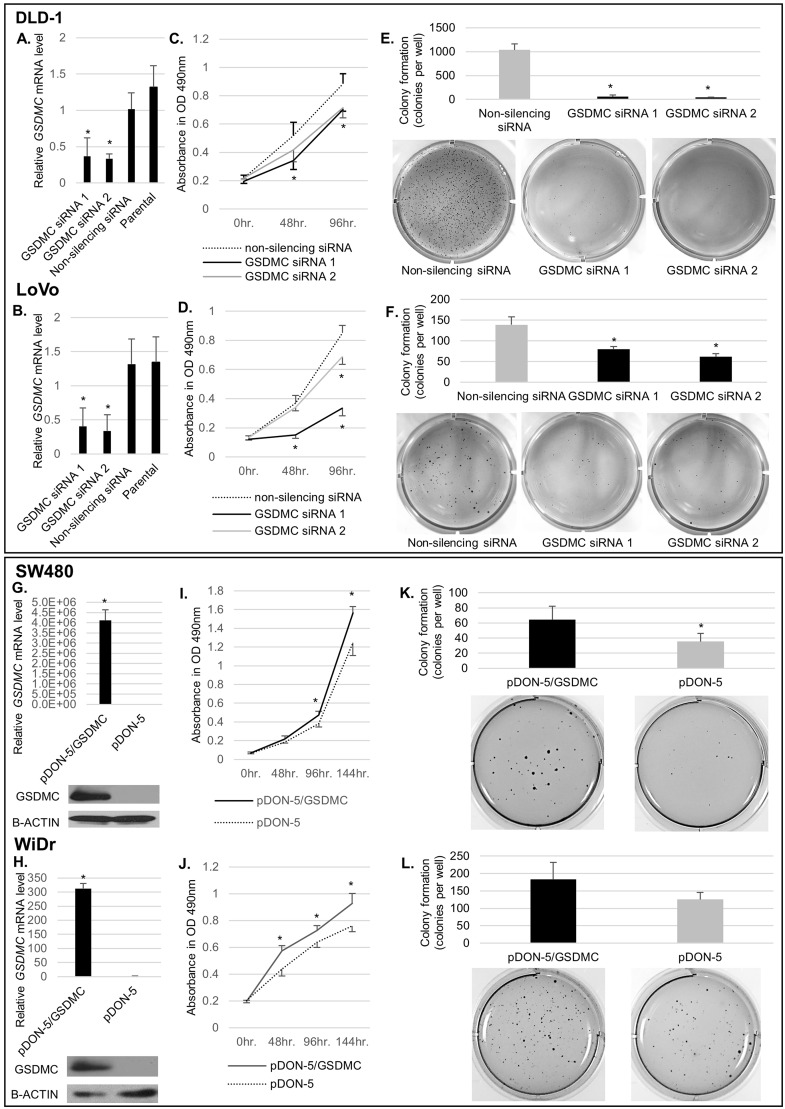
GSDMC promoted tumor growth *in vitro*. (A), (B) Quantitative reverse transcription (qRT)-PCR of the expression of *GSDMC* mRNA after transfection of DLD1 and LoVo cells with siRNAs. (C), (D) MTS assays were used to analyze the effects of *GSDMC* knockdown on cell proliferation in DLD-1 and LoVo cells for up to 4 days. (E), (F) Colony formation assays in DLD-1 and LoVo cells. Upper panel: number of colonies; lower panel: imaging results. Colonies were monitored for up to 11 days after cell seeding on the plates. (G), (H) Upper panel: qRT-PCR; lower panel: western blotting analysis in SW480 or WiDr cells transfected with pDON-5/GSDMC or pDON-5. (I), (J) MTS assays in SW480 or WiDr cells transfected with pDON-5/GSDMC or pDON-5. Cell growth was monitored up to 6 days. (K), (L) Colony formation assays in SW480 or WiDr cells transfected with pDON-5/GSDMC or pDON-5. Those in WiDr cells did not show significant differences (*p* = 0.127). Upper panel: number of colonies; lower panel: imaging results. Colonies were monitored for up to 11 days after cell seeding on the plates. Representative data from three independent experiments are shown. *p < 0.05.

Next, we analyzed the effects of GSDMC expression on proliferative ability by silencing or overexpressing GSDMC in CRC cell lines. DLD-1 and LoVo cells, which had relatively high endogenous expression of *GSDMC* mRNA (S1 Fig), were transfected with two small-interfering RNAs (siRNAs) targeting *GSDMC*. qRT-PCR confirmed that the siRNAs significantly blocked *GSDMC* expression compared with that in cells transfected with nonsilencing RNA (Fig 4A and 4B).

MTS assays revealed that the proliferation of cells transfected with GSDMC siRNA1 and siRNA2 was inhibited compared with that in cells transfected with nonsilencing RNA (Fig 4C and 4D; *p* < 0.05). Furthermore, in colony formation assays, GSDMC silencing resulted in a significant decrease in anchorage-independent growth ability in DLD-1 cells (Fig 4E; GSDMC siRNA1-transfected cells: 57.3 ± 32.3 colonies per well, GSDMC siRNA2-transfected cells: 43.7 ± 2.5 colonies per well, control cells; 1036.7 ± 125.4 colonies per well; *p* < 0.05) and LoVo cells (Fig 4F; GSDMC siRNA1-transfected cells: 80.0 ± 6.4 colonies per well, GSDMC siRNA2-transfected cells: 61.7 ± 7.2 colonies per well, control cells: 138.7 ± 19.0 colonies per well; *p* < 0.05).

Conversely, SW480 and WiDr cells, which exhibited low endogenous expression of *GSDMC* mRNA (S1 Fig), were transfected with a vector encoding human full-length *GSDMC* (pDON-5/GSDMC) or an empty vector (pDON-5). The efficiency of GSDMC overexpression is shown in Fig 4G and 4H. MTS assays revealed that cell proliferation was significantly promoted in cells transfected with pDON-5/GSDMC compared with that in cells transfected with the pDON-5 vector for both cell lines (Fig 4I and 4J; *p* < 0.05). GSDMC overexpression in SW480 cells resulted in a significant increase in anchorage-independent growth ability (Fig 4K; 64.3 ± 17.7 colonies per well versus 35.3 ± 10.7 colonies per well, respectively; *p* < 0.05). GSDMC overexpression in WiDr cells tended to increase anchorage-independent growth ability (Fig 4L; 183.0 ± 48.6 colonies per well versus 125.3 ± 20.8 colonies per well, respectively; *p* = 0.127). These results suggested that GSDMC promoted the tumorigenesis of CRC cell lines.

### GSDMC promoted xenograft tumor growth

**Fig 5:**
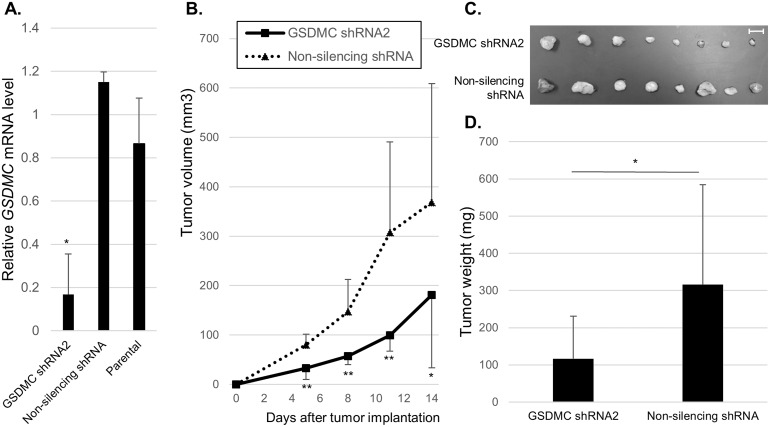
GSDMC promoted xenograft tumor growth *in vivo*. (A) Quantitative reverse transcription PCR was used to analyze the expression of GSDMC in LoVo cells stably expressing *GSDMC* shRNA2 or nonsilencing shRNA. (B)–(D) Effects of GSDMC silencing in LoVo cells on the growth of xenograft tumors in nude mice (n = 8). Cells stably expressing *GSDMC* shRNA2 or nonsilencing shRNA were injected subcutaneously into nude mice. The growth of subcutaneous tumors was measured every 3 days using Vernier caliper, and volume determined by the formula V = 1/2 (length × width^2^). The tumors were then removed from the nude mice and imaged (C). Tumor weights are shown in (D). *p < 0.05, ** *p* < 0.01

To further investigate the importance of GSDMC in colorectal tumorigenesis in an *in vivo* model, we generated LoVo cell lines stably expressing *GSDMC* shRNA2 or nonsilencing shRNA. After confirming the stable silencing of GSDMC by qRT-PCR (Fig 5A), we implanted GSDMC-knockdown cells or control cells into nude mice (n = 8 each). The tumor growth speed in mice inoculated with GSDMC-knockdown cells was significantly slower than that in the control group (Fig 5B). The volumes of tumors removed from mice inoculated with GSDMC-knockdown cells were smaller than those removed from mice inoculated with control cells (Fig 5C). Moreover, tumor weights were significantly reduced in mice inoculated with GSDMC-knockdown cells compared with that in mice inoculated with control cells (Fig 5D; 116.6 ± 114.1 versus 315.8 ± 268.8 mg, respectively; *p* < 0.05). These results indicated that GSDMC played an important role in promoting proliferation in colorectal tumorigenesis *in vivo*.

## Discussion

In this study, we generated a CRC mouse model in which both *Tgfbr2* and *Apc* were inactivated in the colon epithelium; these mutations induce the formation of adenocarcinomas in the proximal colon. We then identified *GSDMC* as a novel gene upregulated by TGFBR2 inactivation using microarray analysis. Further experiments confirmed that *GSDMC* promoted tumor cell proliferation in colorectal carcinogenesis. Therefore, GSDMC may be a promising therapeutic target in patients with CRC harboring *TGFBR2* mutations.

A previously established CRC mouse model of *Tgfbr2* conditional knockout revealed that *Tgfbr2* inactivation promoted the progression of adenomas initiated by *Apc* inactivation to invasive adenocarcinomas with a mucinous component [17]. In human CRC, *TGFBR2* mutations are frequently observed as a 10-bp poly-adenine repeat in exon 3, called the big adenine tract (BAT-RII); the BAT-RII is a microsatellite sequence prone to replication errors, resulting in insertion or deletion of one or two adenines, thereby introducing truncated, nonfunctional TGFBR2 protein. To recapitulate TGFBR2 mutations in a mouse model, we used *Tgfbr2*^*flox/flox*^ mice, which harbor the *Tgfbr2* allele having *loxP* sites that flank exon 2 [26]. In this model, exon 2 of *Tgfbr2* was removed using a Cre-loxP system, resulting in Tgfbr2 protein inactivation. In this study, we generated *CDX2P-G19Cre*;*Apc*^*flox/flox*^;*Tgfbr2*^*flox/flox*^ mice, which exhibited *Tgfbr2* and *Apc* inactivation in the colon epithelium, using previously generated mouse models [22–25]. Subsequent microarray analysis revealed that *Gsdmc4|Gsdmc2* was the most highly upregulated gene between *CDX2P-G19Cre*;*Apc*^*flox/flox*^;*Tgfbr2*^*flox/flox*^ and *CDX2P-G19Cre*;*Apc*^*flox/flox*^ mice.

The *Gsdm* family genes comprise four human genes (*GSDMA*, *GSDMB*, *GSDMC*, and *GSDMD*) and eight mouse genes (*Gsdma1–3*, *Gsdmc1–4*, and *Gsdmd*). These genes were originally named based on their expression profile; *Gsdm* family genes are expressed in a cell and tissue type-specific manner in epithelial tissues throughout the gastrointestinal tract and dermis [35, 36]. Additionally, each *Gsdm* family may be involved in epithelial cell proliferation, differentiation, and apoptosis and may play a crucial role in tumorigenesis in various organs [37]. *GSDMC* was originally identified as melanoma-derived leucine zipper extranuclear factor (*MLZE*), whose cDNA was isolated from a melanoma cell line. The mouse homolog of *GSDMC* consists of four *Gsdmc* clusters, which exhibit amino acid sequence similarity with each other and are located on chromosome 15 (15D1) [36]. Because the expression levels of individual *Gsdmc* cluster genes cannot be distinguished from one another due to crossreactivity of microarray probes, *Gsdmc4|Gsdmc2* was identified as a significantly upregulated gene pair. However, because other *Gsdmc* cluster pairs also tended to be upregulated (as shown in S5 Table), we assumed that *Gsdmc1–4* function as a gene cluster and are upregulated by *Tgfbr2* mutation. Upregulation of *Gsdmc2* and *Gsdmc4* was validated in RNA from mouse tumors using specific primers designed for qRT-PCR analysis.

Expression of *GSDMC* mRNA, which is observed in the epithelium of the esophagus and stomach, is suppressed in gastric and esophageal cancer cell lines, and proliferation assays with gastric cancer cell lines showed that *GSDMC* has cell-growth inhibitory activity, suggesting that GSDMC may function as a tumor suppressor [37]. In contrast, GSDMC is not expressed in the normal epidermis, but is expressed in melanoma specimens; this expression is correlated with the invasiveness and metastatic potential of melanoma cells [38]. In a recent study, chromosomal engineering in mice showed that copy number gains in the *Myc* gene promote tumorigenesis in mammary tumors only if the downstream sequence encompassing *Pvt1*, *Ccdc26*, and *Gsdmc* is also amplified [39], suggesting that GSDMC may play a role in tumor progression. Therefore, the function of GSDMC in carcinogenesis is unclear, and GSDMC may have organ-specific roles. In this study, we investigated the function of GSDMC in CRC. GSDMC was not expressed in normal colonic tissues, but was highly expressed in CRC tissues at both the mRNA and protein levels (Fig 3). Furthermore, silencing of GSDMC led to a significant reduction in proliferation and tumorigenesis in CRC cell lines in vitro and *in vivo*, whereas overexpression of GSDMC enhanced CRC cell proliferation *in vitro*. These results suggested that GSDMC functioned as an oncogene by promoting cell proliferation in colorectal carcinogenesis.

Our studies with the *CDX2P-G19Cre*;*Apc*^*flox/flox*^;*Tgfbr2*^*flox/flox*^ mouse model yielded some observations that warrant more detailed investigations. First, the mechanisms through which TGF-β signaling regulate the expression of GSDMC was not determined despite our challenges with the identification of the promoter region of *GSDMC*, although previous studies have demonstrated that GSDMA in same family members of GSDMC are regulated by TGF-β signaling [40]. However, the available methods are limited in terms of revealing physiological activity in a complex animal [41]. Additionally, in this study, we showed that conditional inactivation of *Tgfbr2* in the context of *Apc* mutation in the colon epithelium results in noninvasive well-differentiated adenocarcinoma, similar to the morphological phenotype observed with *Apc* mutation alone. This observation may be explained by the fact that *Apc* homozygous knockout in *CDX2P-G19Cre* mice induced rapid growth of polypoid tumors, and the mice did not survive for more than 4 weeks. Because *Tgfbr2* inactivation was confirmed by genotyping and immunohistochemical analysis in tumors of *CDX2P-G19Cre*;*Apc*^*flox/flox*^;*Tgfbr2*^*flox/flox*^ mice, the expression of genes influenced by Tgfbr2 inactivation was assumed to change, and such genes were identified by microarray analysis. However, the mice exhibiting distinct morphological characteristics induced by *Tgfbr2* inactivation, such as deep invasiveness and mucinous carcinoma, was a more convincing tool for identifying changes in gene expression induced by *Tgfbr2* inactivation. To achieve this, it may be useful to generate a CRC mouse model having haploinsufficiency of *Apc*, in which tumors develop more slowly and mice live a longer life [42].

*In vitro*, TGF-β signaling has been shown to induce growth arrest by inhibiting the expression of cyclins/cyclin-dependent kinases (CDKs) by inducing the CDK inhibitors p15INK4B, p21Cip1, and p27KIP1 and blocking c-myc induction [43, 44, 45, 46, 47]. However, the expression levels of these TGF-β signaling targets were not significantly altered in our microarray analysis. Notably, these genes were identified as targets of TGF-β signaling by in vitro assays, demonstrating the simple additive effects of treatment with TGF-β family ligands. Furthermore, GSDMC was identified in a CRC mouse model involving the microcirculation/stromal microenvironment, which an in vitro system cannot mimic.

In conclusion, we generated a CRC mouse model in which inactivation of *Tgfbr2* and *Apc* in the colon epithelium induced the formation of adenocarcinomas. This unique model has the potential to improve our understanding of CRC with *TGFBR2* mutations. Subsequent gene profiling analysis revealed that GSDMC was upregulated by *TGFBR2* mutation in CRC. Furthermore, GSDMC promoted tumor cell proliferation in colorectal carcinogenesis and may be a promising therapeutic target for patients with CRC having *TGFBR2* mutations.

## Supporting Information

S1 Fig Quantitative reverse transcription PCR analysis showing the relative expression levels of *GSDMC* mRNAs in eight colorectal cancer cell lines. Bars indicate means + SDs.
(TIF)

S1 Table The sequences of primers (*Mus musculus* and *Homo sapiens*) used for quantitative RT-PCR and sequencing analysis. (XLSX)

S2 Table The sequences of the siRNAs/shRNAs used for GSDMC silencing and those of PCR primers used for GSDMC overexpression. The coding region in *GSDMC* mRNA was cloned by PCR using the indicated primers, and the product was inserted into a retroviral vector.
(XLSX)

S3 Table Gene sets differentially expressed in tumors of *CDX2P-G19Cre*;*Apc*^*flox/flox*^;*Tgfbr2*^*flox/flox*^ mice in comparison with those of *CDX2P-G19Cre*;*Apc*^*flox/flox*^ mice analyed with c4 gene seta in MSigDB using GSEA software and Simple Array Analyzer. (XLSX)

S4 Table Gene sets differentially expressed in tumors of *CDX2P-G19Cre*;*Apc*^*flox/flox*^;*Tgfbr2*^*flox/flox*^ mice in comparison with those of *CDX2P-G19Cre*;*Apc*^*flox/flox*^ mice analyed with c5 gene seta in MSigDB using GSEA software and Simple Array Analyzer. (XLSX)

S5 Table The sets of *Gsdmc* cluster genes whose expression levels were altered in the tumors of *CDX2P-G19Cre*;*Apc*^*flox/flox*^;*Tgfbr2*^*flox/flox*^ mice compared with those of *CDX2P-G19Cre*;*Apc*^*flox/flox*^ mice, as shown in microarray analysis. The expression of other *Gsdmc* cluster gene sets tended to increase, similar to *Gsdmc2|Gsdmc4*, whose expression increased significantly.
(XLSX)
