# Supplementary figures and images for: Gasdermin C Is Upregulated by Inactivation of Transforming Growth Factor β Receptor Type II in the Presence of Mutated Apc, Promoting Colorectal Cancer Proliferation
Source: PLoS One. 2016 Nov 11;11(11):e0166422. doi: 10.1371/journal.pone.0166422 (PMC5105946; doi:10.1371/journal.pone.0166422)

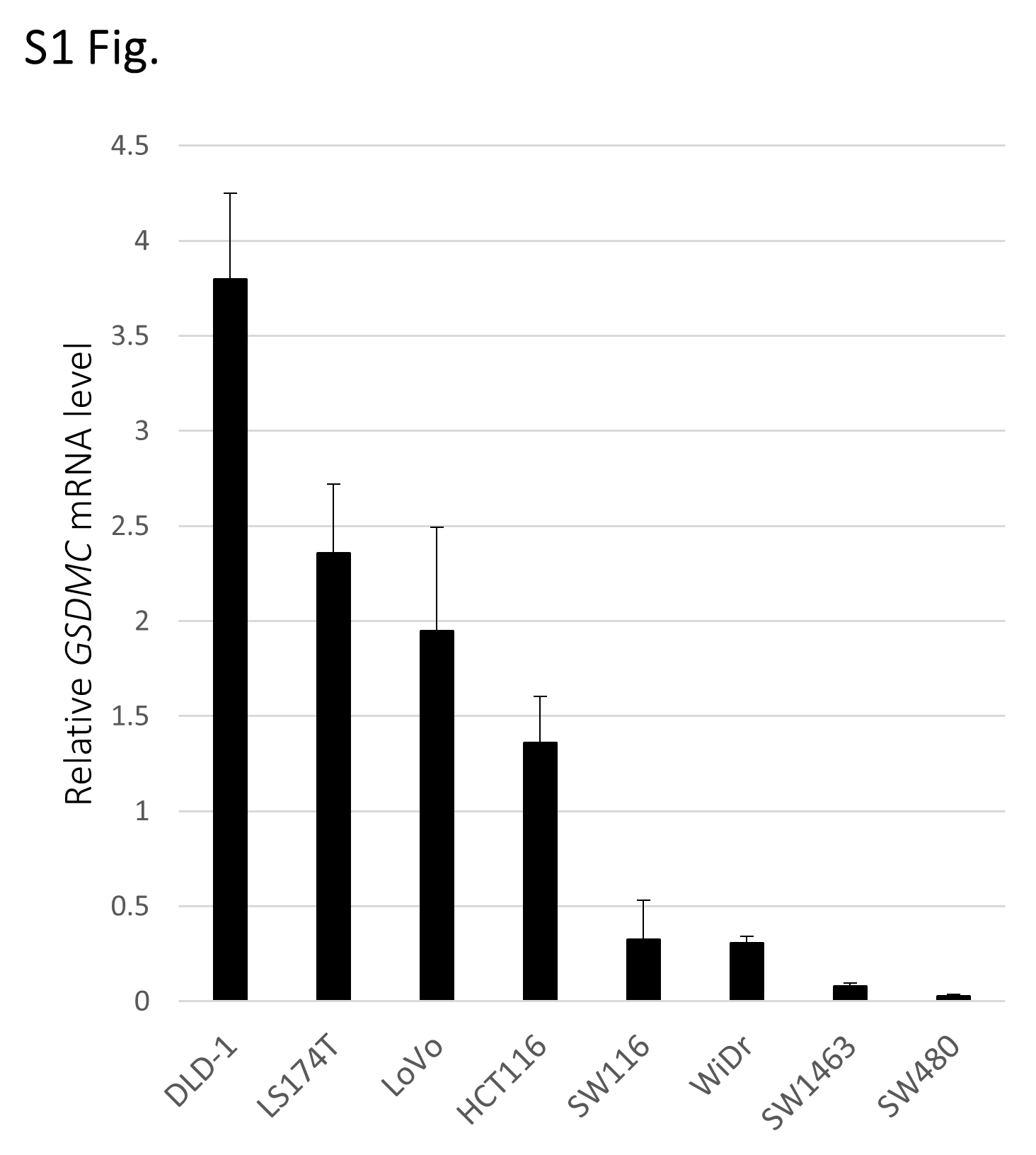

Supplement: S1 Fig — Bars indicate means + SDs. (TIF) [file pone.0166422.s001.tif]
